# Microtubule Dysfunction: A Common Feature of Neurodegenerative Diseases

**DOI:** 10.3390/ijms21197354

**Published:** 2020-10-05

**Authors:** Antonella Sferra, Francesco Nicita, Enrico Bertini

**Affiliations:** Unit of Neuromuscular and Neurodegenerative Disorders, Genetics and Rare Diseases Research Division, Bambino Gesù Children’s Hospital, IRCCS, 00146 Rome, Italy; francesco.nicita@opbg.net (F.N.); enricosilvio.bertini@opbg.net (E.B.)

**Keywords:** microtubules, neurodegeneration, microtubule dysfunctions, microtubule-targeting compounds

## Abstract

Neurons are particularly susceptible to microtubule (MT) defects and deregulation of the MT cytoskeleton is considered to be a common insult during the pathogenesis of neurodegenerative disorders. Evidence that dysfunctions in the MT system have a direct role in neurodegeneration comes from findings that several forms of neurodegenerative diseases are associated with changes in genes encoding tubulins, the structural units of MTs, MT-associated proteins (MAPs), or additional factors such as MT modifying enzymes which modulating tubulin post-translational modifications (PTMs) regulate MT functions and dynamics. Efforts to use MT-targeting therapeutic agents for the treatment of neurodegenerative diseases are underway. Many of these agents have provided several benefits when tested on both in vitro and in vivo neurodegenerative model systems. Currently, the most frequently addressed therapeutic interventions include drugs that modulate MT stability or that target tubulin PTMs, such as tubulin acetylation. The purpose of this review is to provide an update on the relevance of MT dysfunctions to the process of neurodegeneration and briefly discuss advances in the use of MT-targeting drugs for the treatment of neurodegenerative disorders.

## 1. Introduction

Microtubules (MTs) are structurally and functionally important components of the cell cytoskeleton, providing most of the spatial organization in the cell, and regulating different cellular functions such as intracellular trafficking, cell division, and organelle positioning.

They are characterized by a great chemical and functional heterogeneity, and are thus subjected to highly dynamic regulatory mechanisms that modulate MT composition, tubulin chemical modification, and MT association with other proteins. The result of these regulations is the generation of MT arrays with different morphology and degrees of stability that are able to adapt to specific functions in accordance with the cell context.

This modulation is particularly important in neurons, whose structures and functions strongly depend on the correct functioning of MTs. It is therefore not surprising that MT dysfunctions can be the cause or can contribute to the onset of neurodegenerative processes. Specific mutations in tubulin genes, dysfunctions of MT-associated proteins (MAPs), and alterations in the levels of tubulin PTMs are indeed associated with different forms of neurodegenerative diseases. This discovery has recently allowed the development of compounds aimed to counteract neurodegeneration by restoring the correct functioning of MTs. Although, recently, MT-oriented approaches currently represent one of the most promising strategies in the field of neurodegenerative diseases as several works have demonstrated that these compounds are effectively able to prevent or partially rescue MT dysfunctions in different animal and cellular models and that some of them have been translated into clinical trials. These results lead us to believe that in the near future, more preclinical approaches will be translated to patients, improving survival and their quality of life.

## 2. Microtubule Structural and Functional Complexity

**Figure 1:**
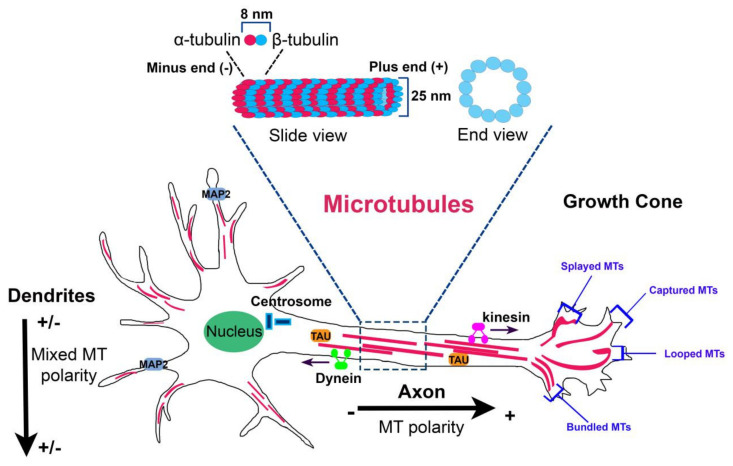
Microtubule organization in neurons. Microtubule (MT) organization is tightly regulated in the different neuronal compartments. In axon, MTs form stable, polarized bundles with uniform polarity orientation, exposing their plus minus ends away from the cell body. In proximal dendrites, MTs are organized in antiparallel bundles oriented with their plus ends pointing away or toward the soma. In the growth cone, MTs adopt four characteristic distributions: splayed, captured at the cortical matrix, looped, and bundled. At the top, MT structure (slide view and end view) is shown.

MTs are composed of heterodimers of α–β-tubulin that align head to tail to form linear protofilaments which associate laterally to form a hollow, polar cylinder [1]. The directional alignment of polarized α–β heterodimers into MTs make the structures intrinsically polarized because of the presence two distinct ends: a minus end that exhibits an α-tubulin subunit and the opposite plus end that exposes a β-tubulin subunit (Figure 1).

In a solution of purified tubulin, MT elongation can occur to both ends [2]. In cells, MT growth is significantly more rapid at the plus end and occurs through the addition of α–β tubulin heterodimers in which each subunit of β-tubulin binds a GTP molecule. After heterodimer addition, the GTP is hydrolyzed to GDP and prolonged exposure of a terminal GDP at the α–β heterodimer of the plus end leads to MT depolymerization [3].

Within the cell, MTs do not reach a steady state length but undergo continual assembly and disassembly; the co-existence of growing and shrinking MTs in the same conditions is known as “dynamic instability”. Conversion from growth to shrinkage is defined “catastrophe”, whereas the switch from shrinkage to growth is called “rescue”. This “non equilibrium” in MT population is critical for several cell functions, such as cell division or during or the spatial arrangements of the cell [4]. 

MTs also undergo “treadmilling”, a dynamic process in which the plus end of the filament grows in length while the other one shrinks due to the removal of tubulin molecules bound to GTP from the minus end that travel to the plus end of the same MT [5].

In most cell types, MTs are nucleated at the centrosomal region in the MT-organizing center (MTOC), where a previously formed γ-tubulin ring complex provides a base for the start of filament extension [6]. The γ-tubulin ring complex has been proposed to form a ring-like template that binds the α-tubulin subunit exposed at the MT minus end. Thus, MTs grow with a defined polarity, with their minus ends anchored on the ring to γ-tubulin subunits and the plus end of the MT extending into the cytoplasm [7].

MTs constitute a heterogeneous and dynamic filament network with great chemical and functional complexity. 

This complexity is produced by three regulatory mechanisms: (i) differential incorporation of alternative isotypes of α and β tubulin into MTs; (ii) PTMs of these isotypes; (iii) MT interaction with MAPs. These mechanisms do not act separately but can influence each other. Each of these will be briefly discussed below and in the specific context of neuronal cells.

In many organisms, both α and β tubulins are encoded by multiple genes at different loci [8,9]. Most of them, such *TUBB2A*, *TUBB2B*, and *TUBA1A*, *TUBA1B*, *TUBA1C*, are clustered in the genome and are presumably the products of genomic duplications [10]. In vitro experiments have shown that tubulin isotypes have different effects on MT dynamics [11,12,13,14], suggesting that they can differentially regulate the MT network in vivo. It was assumed that tubulin isotypes can assemble into discrete MT species that carry out unique functions [15]. This concept, known as the “multi tubulin hypothesis”, has long been quite controversial. It was demonstrated that all tubulin isotypes freely co-polymerize into heterogeneous MTs and that different α- and β-tubulin isotypes in fungi were functionally interchangeable [16,17]. However, several findings have shown that highly specialized MTs, such as ciliary axonemes [18,19], neuronal MTs [20,21], and MTs of the marginal band of platelets [22,23] depend on specific tubulin isotypes. Axonemal MTs of Drosophila olfactory neurons are highly enriched in β1- and β4-tubulins [24]. β6-Tubulin, instead, is essential for the formation of the platelet marginal band and the loss of this isoform results in thrombocytopenia [23]. Studies in *Drosophila melanogaster* have shown that specific tubulin isotypes are not functionally equivalent and possess distinctive properties depending on cell type and specific stages of development [25,26]. Hoyle and colleagues have demonstrated that in Drosophila male testes, the function of β2-tubulin is not rescued by the expression of β3-tubulin, and that the latter acts in a dominant-negative manner when co-expressed at levels exceeding 20% of the total tubulin. This finding indicates that while β2-tubulin regulates generic functions in Drosophila male testes, β3-tubulin has been evolved to ensure different MT functions in fly [24]. Similarly, in mouse *tubb3^−^*^/−^ neurons the replacement of β3-tubulin by other β-tubulin isoforms cannot overcome its requirement in peripheral nerve regeneration, suggesting that the functions of the different β-tubulins are not interchangeable in neurons [27]. 

Tubulin isotypes also seem to directly determine MT structure by defining its protofilament numbers. In vitro experiments have shown that dimers of α1B-tubulin and β3-tubulin form typical MTs composed by 13 protofilaments, while heterodimers of α1β-tubulin and β2B-tubulin preferentially assemble into MTs composed by 14 protofilaments [28]. Moreover, in touch receptor neurons of *Caenorhabditis elegans*, MEC-12 α-tubulin and MEC-7 β-tubulin assemble to form 15 protofilaments [29,30,31]. Interestingly, MEC-12 α-tubulin is the only tubulin subjected to acetylation in *C. elegans*. This modification seems to be crucial for the assembly of these characteristic 15-protofilament MTs and the absence of αTA1, the enzyme that catalyzes MEC-12 α-tubulin acetylation, causes the formation of MTs with a lower protofilament number in *C. elegans* sensory neurons [32].

This finding introduces another important regulatory mechanism of MTs, which is that of tubulin PTMs. Tubulin isotypes are indeed further diversified through a large number of chemically induced PTMs. Some of these, such acetylation, are ubiquitous protein modifications, while others, such polyglutamylation, even appear to be unique to tubulins [33]. Almost all PTMs are reversible modifications and with the exception of acetylation, that occurs on the luminal surface of MTs, they take all place on the carboxy-terminal domain of tubulins [34]. Different PTMs occur concurrently on MTs; they can be scattered or concentrated in distinct domains, and depending on their localization and combination, create different structural patterns that regulate specific MT functions.

Most tubulin PTMs occur on axonemes, the MT-based core structures of eukaryotic cilia and flagella, and alterations of their levels have been found to impair the proper maintenance and functioning of these structures [35,36,37,38].

Analogous to the model of the “histone code” on chromatin, it was proposed that PTMs on the MT surface “write” a biochemical “tubulin code” that is “read” by specific proteins [39]. 

The specialized functions of MT are also determined by a large number of protein partners collectively known as MAPs. MAPs were first discovered as proteins that bind and stabilize MTs. The huge number of MAPs identified in the last years has indeed revealed a more complex picture of their functional properties. They constitute a class of heterogeneous regulators which perform the most disparate functions within the cells, such as the modulation of MT stability and dynamics, the directional transport of cargo, or the regulation of MT nucleation.

Thus, each of these three regulatory mechanisms adds a level of complexity to MT properties and, together with the others, establishes and regulates the specialized behavior of MTs. The result of these regulations is the creation of spatial and temporal patterns of MT network within the cell. This modulation is particularly important in neurons: the precise organization of MTs, including their composition, modification, spacing, and interaction, is essential for the correct morphology and function of these cells.

## 3. Microtubule Organization in Neurons

Neurons are highly compartmentalized and polarized cells whose structure and functions rely on MTs. Neuronal MT arrays are very heterogeneous and consist of single MT with different lengths and stability [40,41,42,43]. Long MTs have a dual function: they act as structural backbone that opposes to axon and dendrite retraction and as tracks for the transport of membrane-bound vesicles and organelles [44]. Indeed, short MTs are highly dynamic elements that move along axons, where they act as MT nucleation sites and sources for the generation of free α- and β-tubulin subunits that are used for the elongation of growing MTs [45,46].

Differently from non-neuronal cells that present uniformly oriented MTs anchored at the MTOC, neuronal MTs are discontinuous along axonal and dendritic processes. In young neurons, MTs are nucleated at the centrosome as short polymers that are rapidly released and transported into neurites [47]. The release of MTs from the MTOC is critical for the axonal growth and inhibition of katanin, the enzyme that severs MTs from the centrosome, suppresses axonal elongation [48]. 

From the early stage of maturation of developing neurons, centrosomes lose their function as MT-organizing centers and MTs are nucleated through non-centrosomal mechanisms, such as at the Golgi outposts [49,50] or from the lateral surface of pre-existing MTs [51,52].

The organization of neuronal MTs is tightly regulated in axons and dendrites. In axons, MTs have uniform polarity orientation, with the plus ends are directed away from the cell body [53], while in proximal dendrites, MTs are organized in antiparallel bundles oriented with their plus ends pointing away or toward the soma [54] (Figure 1). This orientation is, in part, regulated by kinesin and dynein molecular motors that transport nucleated MTs in the different neuronal compartments sliding along existing MTs [55,56,57]. Dynein and kinesin depletion disrupts the axonal bidirectional transport of plus end and minus end out MTs and the dendritic transport of minus end out MTs, respectively, resulting in impaired MT orientation in these compartments [55,57,58,59,60]. Preservation of the distinct patterns of MT polarity in axons and dendrites ensures the maintenance of the morphological and structural differences between these compartments.

For example, dendrites are restricted to small regions around the cell body while axons can extend to great distances. This happens because vesicular organelles that expand the cell membrane have a unidirectional vector to the tip of the growing axon via their plus end distal MTs [47]. MT orientation in axon and dendrites also affect their organelle compartmentalization. Dendrites contain cellular organelles, such as endoplasmic reticulum, polyribosome, and Golgi outposts that are rare in axons [61]. This occurs because these organelles are transported by minus end directed motor proteins that move from the soma to the minus end out MTs, which are exclusively present in dendrites [62].

At the end of the axon, MT arrays extend deep into the growth cone when bundles splay and adopt characteristic distributions (splayed, captured, looped, and bundled) [63,64] (Figure 1). The major function of MTs into growth cone is that to drive its directed movement; MT distribution on one rather than another side into the growth cone decides where it will turn [63].

## 4. Neuronal Microtubules: Regulative Mechanisms

### 4.1. Incorporation of Specific Tubulin Isotypes

Neuronal MTs rely on specific tubulin isotypes. Except for some of them that have been intensely characterized during mouse brain development, mainly through experiments of RNA in situ hybridization and most recently through the generation of transgenic mouse models, the spatial and temporal expression patterns of neuronal tubulin isotypes are still not completely understood, principally due to the difficulties in tracking protein expression due to their high homology.

Among the different tubulin isotypes expressed in the brain, β3-tubulin (encoded by *TUBB3* human gene and *tubb3* mouse gene) is considered a specific marker for neuronal MTs and has been largely characterized [65,66]. Its expression is primarily limited to post-mitotic neurons in development and adulthood [65] and is greatest during the periods of axon guidance and maturation [67]. During the embryonic stages, it is earlier expressed in both peripheral and central nervous system (at E9.0–E9.5) and later in the forming neocortex and retina (at E10.5–E12.5). Indeed, during the postnatal period, β3-tubulin is expressed in neurons of the layer II–VI and in the inner granular cell layer and Purkinje cells of the cerebellum. β3-tubulin expression is also maintained in the postnatal period in regions of the central nervous system where adult neurogenesis occurs, such as cells of the olfactory bulb and dentate gyrus [66].

Purified MTs assembled from β3-tubulin are less resistant to depolymerization and spend less time in paused states than those composed of β2- and β4-tubulin or a mixture of all three isotypes [68]. These findings suggest that neuronal MTs are more dynamic, a concept that was initially hypothesized after the observation that brain MT depleted of β3-tubulin showed an increased assembly speed in vitro [69]. According to these in vitro findings, the lack of β3-tubulin in *tubb3* knockout mouse model decreases the dynamics of MTs in growth cones and reduces the neurite outgrowth rate in vivo [27]. Depending on the causative mutation, *TUBB3* mutations are associated with a broad range of neurological conditions, including malformations of cortical development (i.e., polymicrogyria, gyral disorganization, basal ganglia anomalies, abnormal corpus callosum, hypoplastic brainstem, dysplastic cerebellar vermis), a congenital cranial dysinnervation disorder known as congenital fibrosis of the extraocular muscles type 3 (CFEOM3), and axonal sensorimotor neuropathy [70,71,72,73,74]. 

Other tubulin isotypes, such as α-tubulin1A (encoded by human *TUBA1A* and mouse *tuba1a*), α8-tubulin (encoded by human *TUBA8* and mouse *tuba8* ), β-tubulin2B (encoded by human *TUBB2B* and mouse *tubb2b*), β-tubulin2A (encoded by human *TUBB2A* and mouse *tubb2a*), the class I β-tubulin (encoded by *TUBB* human gene and *tubb5* mouse gene), α-tubulin4A (encoded by human *TUBA4A* and mouse *tuba4a*), and β-tubulin4B (encoded by human *TUBA4B* and mouse *tuba4b*) are reported to be expressed in brain. 

The neurological disorders caused by mutations in genes encoding these tubulins as well as in *TUBB3* are known as “tubulinopathies” [75,76,77]. The disease spectra associated with these different tubulin isotypes are overlapping but distinct, reflecting functional specification of the tubulin multigene family.

Among the β-tubulin isotypes, class I β-tubulin is the highest expressed in developmental mouse brain [78]. In situ hybridization and immunocytochemical experiments, conducted respectively on the brain tissue of wild type and *tubb5* transgenic mice, have revealed predominant expression of this tubulin in neuronal progenitors and post-mitotic neurons during fetal cortical development [78]. Perturbation of *tubb5* impairs differentiation of neuronal cortex neurons and the maturation of dendritic spines in mouse [79]. Patients with *TUBB* mutations exhibit neurological features including microcephaly, polymicrogyria, and dysmorphic basal ganglia which can occur concurrently to the “Michelin tire baby syndrome”, characterized by congenital symmetrical circumferential skin creases and facial abnormalities [78,80,81,82,83].

Similarly to class I β-tubulin, β-tubulin 2B is highly expressed in progenitors and post-mitotic neurons during mouse cortical development [84]. Its expression decreases in postnatal cortex and persists only in a population of post-mitotic neurons in 8-week-old retina [84]. Mutations in *TUBB2B* principally cause polymicrogyria, pachygyria, microcephaly, and axon guidance defects [85,86,87,88,89]. 

Much less is known about the spatial and temporal expression of β-tubulin2A. This tubulin isotype represents ~30% of all β-tubulins in human brain [90]. Using real-time PCR experiments, Breuss and colleagues have demonstrated lower expression of *tubb2a* during brain development, which reached its highest level at E14.5, E16.5, and around the time of birth [78]. This finding suggests that this tubulin carries out relatively minor functions during brain development, in accordance with the mild brain phenotypes observed in patients harboring *TUBB2A* mutations [91,92,93] and the recent finding that the loss of *tubb2a* leads to relatively mild cortical malformations in mice [10]. Accordingly, we have recently described a novel *TUBB2A* mutation associated with a phenotype presenting peripheral sensory motor polyneuropathy without cortical malformation [94].

It is captivating to hypothesize that expression of *TUBB2A* in brain may be maintained, if not even higher in the postnatal period, as reported for *TUBA4A*, which is associated with a later age disease phenotype without presenting brain malformations.

*TUBA4A* brain expression levels have been reported to dramatically increase (>50-fold) with age. Further, RNA-Seq experiments have shown that *TUBA4A* is the highest β-tubulin expressed in human motor cortex after birth, reaching its maximum expression between 4 months and 1 year and at 8, 19, and 23 years after the birth [90]. This expression pattern may explain why, differently from congenital tubulin mutations, mutations in *TUBA4A* cause amyotrophic lateral sclerosis (ALS), a late-onset disease characterized by adult-onset upper and lower motor neuron degeneration [95].

Much less is known about *TUBB4A* expression data. In human brain, Hersheson and colleagues [96] have found that this gene has the highest expression in the cerebellum, followed by putamen and white matter. Furthermore, similar to *TUBA4A*, expression of *TUBB4A* increases over time in human motor cortex, reaching its maximum expression at 11 and between 19 and 30 years [95]. Interestingly, mutations in this gene are responsible for the torsion dystonia type 4 (DYT4) [96,97], an adult-onset generalized dystonia (also known as “whispering dystonia”). Mutations in *TUBB4A* are also associated with hypomyelination and atrophy of the basal ganglia and cerebellum (H-ABC), a rare hypomyelinating disease of infancy and childhood [98,99,100], and with an allelic clinical variant of congenital or childhood-onset hereditary spastic paraplegia [101,102,103].

By contrast, *TUBA1A* is the most prevalent α-tubulin gene expressed in neurons [75,104,105]. Expression studies on its mRNA have shown that that this isotype is the most prevalent α-tubulin expressed in embryonic nervous system, representing more than 95% of total α-tubulin mRNA [106,107], consistent with its pathogenic role in migration disorders such as pachygyria, lissencephaly, and polymicrogyria [104,108,109,110,111,112,113,114]. The loss of *Tuba1a* in mouse is perinatal lethal and leads to significant forebrain dysmorphology [10]. 

Lastly, TUBA8 represents an atypical tubulin isotype. It is the most divergent α-tubulin isotype, showing a protein sequence very similar to that of mouse Tuba8 and higher than the other human α-tubulins [115]. It is expressed during brain development, displaying an expression pattern similar to that of *tubb2b* in the cortical plate and subplate at E15.5–E16.5, and in the postnatal period where it is highly expressed in the cortical layers II–III and V and in the subplate. Its high expression in post-migratory neurons suggests a role for tuba8 in local adjustments of cell position after arrival in the cortical plate [116]. Differently from all tubulin mutations that present an autosomal dominant inheritance, *TUBA8* mutations have an autosomal recessive inheritance and result in polymicrogyria and optic nerve hypoplasia [116].

In conclusion, the complex expression patterns of each of these genes reveals a specific function of tubulin isotypes, in particular, neuronal cell types at specific stages of brain development and during the postnatal period; understanding the exact role of each tubulin isotype in brain still requires further efforts and may be able to explain the variability of neurological phenotypes associated with this class of genes.

### 4.2. Tubulin PTMs

More than that of any other cell type, neuronal MTs are enriched in a variety of PTMs. PTMs differ between neuronal compartments and vary during neuronal differentiation, leading to the widely accepted hypothesis that specific patterns of PTMs are accumulated at a specific stage of neuron maturation and locally control MT functions.

We will focalize on four PTMs, which are acetylation, tyrosination, detyrosination, and polyglutamylation of tubulin, which have received great attention in recent years, particularly in the context of neuronal cells.

#### 4.2.1. Acetylation

With the exception of TUBA8, all α-tubulin isotypes undergo acetylation on lysine 40 (K40), a residue located in the lumen of MT. This is a reversible PTM catalyzed by the tubulin acetyltransferase αTAT1 [117], and is removed by the histone deacetylase 6 (HDAC6) and Sirtuin-2 (SIRT2) [118,119].

αTAT1 preferentially catalyzes the acetylation of MT in its polymerized state; this finding has opened the question of how this enzyme reaches the K40 inside the lumen of the MT. Currently, the most reliable models are that αTAT1 enters inside the MT lumen via irregularities in the MT structure [120,121] or via the opened MT ends [122]. The switching of protofilament numbers and the loss of tubulin dimers from MTs support the first model; different studies have, indeed, proposed that αTAT1 may use these transient defects to locally enter inside MTs and catalyze the acetylation of K40 [121]. The second and most accepted model is instead supported by recent studies which have shown that αTAT1 binds with high affinity to the MT opening ends [122]. Moreover, MT opening ends directly contact different cellular structures enriched in αTAT1, such as focal adhesions or clathrin-coated pits, facilitating entry of the enzyme [123].

Tubulin acetylation has been proposed to stabilize long-lived MTs, preventing their breakage induced by mechanical stress [124,125]. MTs of mechanosensory neurons, which experience repeated mechanical stresses, are typically highly acetylated [124]. Elimination of MT acetylation by either mutating K40 or deleting the gene encoding αTAT1 impairs touch sensitivity [124]. High levels of acetylated α-tubulin have also been observed in cells derived from Wallerian degeneration slow mice, which are resistant to paclitaxel-mediated axonal degeneration [126,127].

In vitro experiments have shown that acetylation of tubulin enhances MT flexibility and confers resilience against deformations induced by repeated mechanical stresses, possibly by weakening inter-protofilament interactions and making MTs more pliable during bending [125]. Thus, in contrast to other PTMs that, as we will see, act through the recruitment of MAPs, intraluminal acetylation directly regulates MT structure [125].

**Figure 2:**
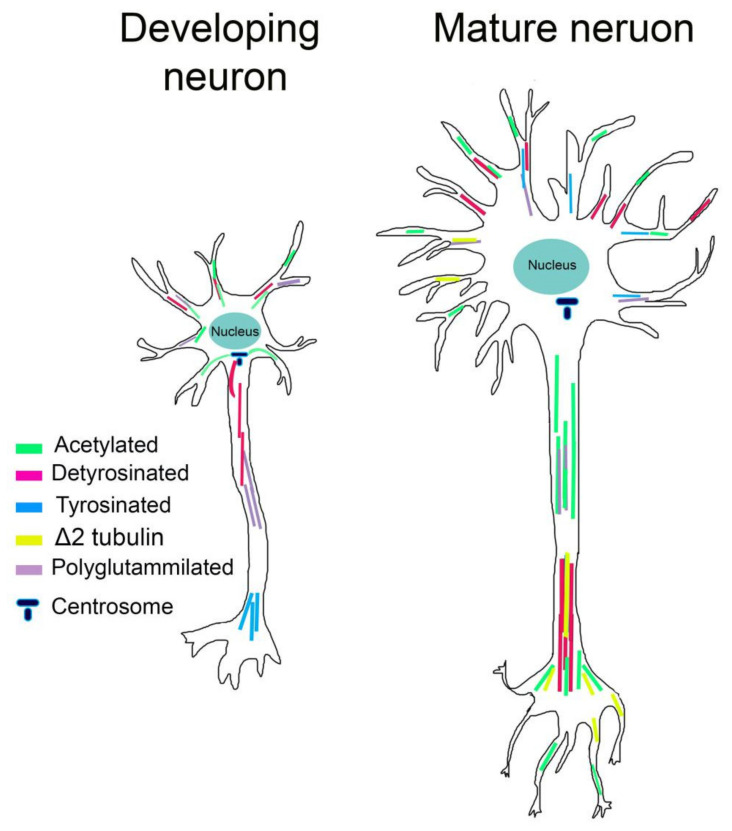
The distribution of tubulin post-translational modifications (PTMs) differs in neuronal compartments and during neuronal differentiation. In young neurons, polyglutamylation and detyrosination are elevated in the growing axon. Acetylation is localized in the cell body connecting neurite outgrowth, while tyrosination is located in distal region of axons contiguous with the growth cone. In mature neurons, acetylation increases in the proximal site of the axon and presynaptic regions. Detyrosinated and Δ2 tubulin levels are relatively high in differentiated dendrites and axons.

In neurons, acetylated MTs are not uniformly distributed. In mature neurons, acetylated MTs are present in lower amounts in the cell body and growth cone and are enriched at the proximal site of the axon, controlling axon branching [128]. Acetylated MTs are also observed in the distal axon and presynaptic regions, consistent with the increase in stable MTs at stable neuronal connections [129] (Figure 2). Instead, in young neurons, acetylated MTs localize to the proximal site of the neurite and in the cell body, connecting neurite outgrowth, while they are absent in the growth cone and in lower amounts in minor neurites [129] (Figure 2). It has likewise been shown that acetylation of MTs increases the recruitment of molecular motors [130,131]. In neuronal cells treated with HDAC inhibitors that increase MT acetylation, the kinesin-1-mediated transport into neurite tips is improved [130,132]. Biochemical experiments have shown that kinesin-1 selectively binds acetylated MTs; however, considering the lumen localization of K40, how this PTM influences kinesin binding is still unclear. It has been recently proposed that acetylated MTs, being enriched in high curve regions, can expose a greater number of available kinesin-binding sites and enhance kinesin run lengths [133].

#### 4.2.2. Detyrosination and Tyrosination

The detyrosination–tyrosination cycle of α-tubulin is the best characterized tubulin modification. These modifications occur in α-tubulin isotypes and consist of the addition and removal of a C-terminal tyrosine residue. With the exception of TUBA4A and TUBA8, that present a C-terminal phenylalanine, all α-tubulin isotypes expose a C-terminal tyrosine residue, which can be removed by detyrosination and re-added by tyrosination [134]. Thus, detyrosination is the initial step that triggers the detyrosination–tyrosination cycle. Tyrosination is catalyzed by the tubulin tyrosine ligase (TTL), while the carboxypeptidase that catalyzes detyrosination has not yet been discovered [33]. Detyrosinated tubulin can be further converted to Δ2-tubulin through deglutamylation of the penultimate glutamic acid residue. Removal of the penultimate glutamic acid residue is irreversible, resulting in the exclusion of Δ2-tubulin from the reversible cycle of detyrosination–tyrosination [135]. Multiple enzymes are capable of removing the penultimate glutamate residue to create Δ2-tubulin, including all six cytoplasmic carboxypeptidases (CCP1–6) [136,137,138,139]. 

In cultured cells, tyrosinated α-tubulin is observed in labile, newly formed MTs whereas detyrosinated tubulin is enriched in stable MT with long turnover [140,141]. In cells with very long-living MTs, detyrosinated tubulin is finally converted into Δ2-tubulin.

Tyrosinated MTs are primarily observed in developing neurons, where they are located in the distal region of axons, contiguous with the growth cone and in the cell body. Detyrosinated MTs are instead distributed in the growing axon and minor neurites of young neurons. Moreover, levels of detyrosinated MTs are high in the axon shaft and in differentiated neurites of mature neurons, while Δ2-tubulin is restricted to very stable MTs and seems to occur during the final stages of neuronal differentiation [129,141,142] (Figure 2).

#### 4.2.3. Polyglutamylation

Polyglutamylation consists of the addition of one or more glutamate residues to the carboxy-terminal tail of both α- and β-tubulin [143,144,145,146].

The addition of strings of glutamines on tubulins consist of two steps: the initiation step, during which the first residue of glutamate is added on the carboxy-terminal domain of tubulin, and the elongation step that leads to the formation of a polyglutamyl side chain [144].

This PTM is catalyzed by nine members of the TTLL family (TTL1, -2, -4, -5, -6, -7, -9, -11, and -13), each of which exhibits a preference for the tubulin isoform (α *vs* β) and for the initiation or elongation steps. TTLL7 preferentially polyglutamylates β-tubulin isoforms while TTLL5 and -6 prefer α-tubulin as a substrate [145]. Moreover, TTLL1, -4, -5, and -7 catalyze the addition of the first residue of glutamate, while glutamate chain elongation is mediated by TTLL6, -9, -11, and -13 [145].

Polyglutamylation is a reversible PTM, and the removal of glutamate residues is catalyzed by CCP enzymes 1, 4, 5, and 6 [137,138].

Although the mechanistic role of polyglutamylation in neurons is still unclear, different studies have reported that polyglutamylation can affect MAPs interactions. The clearest examples come from MAP1, MAP2, tau, and the severing enzymes spastin and katanin [147,148,149,150,151]. Affinity of MAP1B, MAP2, and tau increase progressively for tubulins bearing 1–3 glutamyl units while decreasing for tubulin having longer chains [149,150]. Spastin and katanin are instead preferentially activated by long glutamate side chains [147,148,151]. In addition, this PTM is spatially and temporally regulated in neurons. Glutamylation of α-tubulin is abundant in young neurons whereas glutamylation of β-tubulin increases during postnatal development [152]. Moreover, polyglutamylated α-tubulin is enriched within axons while polyglutamylated β-tubulin accumulates in MAP2-enriched subcellular compartments, where it is required for the growth of MAP2-positive neurites [153] (Figure 2).

### 4.3. Interaction with MAPs

Both the function and organization of MTs, especially neuronal MTs, depends on different MAPs.

Based on their mode of action, MAPs can be roughly divided into five groups. The first group contains the motile MAPs, which use the energy of ATP hydrolysis to walk along MTs. They display unidirectional movement along MTs and include kinesins that move toward the plus end, and are responsible for the anterograde transport, and dyneins that move toward the minus end of MTs and are responsible for the retrograde transport [154]. The second group is that of the MT end-tracking proteins (TIPs) which are distinguished by their specific accumulation at the plus ends (+TIPs) or minus ends of MTs (−TIPs). +TIPs typically target growing but not shrinking MT plus ends where they act mainly as MT-stabilizing factors or link MT ends to various cellular structures, such as the cell cortex or kinetochore [155]. Indeed, −TIPs include protein factors that recognize MT minus ends and regulate MT nucleation, stability or interactions with cellular partners [156].

The third group includes MT-severing enzymes, such as katanin and spastin, that generate internal breaks into MTs [151], while the fourth group comprise MT nucleators, such as the γ-tubulin ring complex which directly modulates MT numbers and nucleation [157] and MT-anchoring proteins, such as the centrosomal proteins ninein and Kif3a that regulate MT aster anchoring [158,159].

Lastly, the fifth group includes the structural MAPs that bind the MT lattice and stabilize or crosslink MTs [160]. These MAPs are highly expressed in the brain and show distinct distribution and expression patterns [161]. Performing such disparate functions, MAPs constitute one of the most complex class of regulators and compensation mechanisms exist among them, as demonstrated by the viability and the absence of a strong phenotype in MAP knockout mice [162,163,164,165,166,167].

The structural MAPs are probably the most intensely studied in the context of neurodegeneration. They are classified in two categories, the type I (MAP1a/1b family) and the type II (MAP2/tau family), with MAP2 and tau the major MAPs expressed in the central nervous system [168].

Both MAP2 and tau exist as alternative spliced isoforms sharing a conserved carboxy-terminal domain containing MT-binding repeats which are involved in the binding of MTs, and an amino-terminal projection domain of varying size [169]. They are natively unfolded molecules which adopt specific conformations upon binding to their targets [170]. MAP2 family is composed of two high-molecular-weight proteins and two low-molecular-weight proteins which are developmentally regulated in brain [169,171,172,173,174,175,176].

Tau has six spliced isoforms [170,171] that differ for the presence of three MT-binding repeats or four MT-binding repeats. In addition, these isoforms are developmentally regulated in brain [177,178,179,180].

Both tau and MAP2 bind longitudinally along the outer ridges of MT protofilaments and exhibit MT-stabilizing activities [181].

Tau is mainly found in axon of neurons where it promotes MT assembly and bundle formation [168,182,183,184]. In vitro experiments have demonstrated that tau promotes tubulin assembly and ring formation [185]. It was also found to increase the time that MTs spent in the pause state, stabilizing MTs in the steady state of assembly [186]. Panda and colleagues showed that a low ratio of tau to tubulin can decrease the treadmilling rate of MTs by 70% in vitro [187]. This is considered one of the principal mechanisms through which tau stabilizes axonal MTs, which are particularly susceptible to treadmilling since their minus ends are not anchored to the centrosome. 

Moreover, it was demonstrated that by binding to the interface between the tubulin dimers, tau controls the spacing between MTs in the bundle via its projection domain [188,189]. Upon binding, tau also induces a local distortion in the protofilament, changing the spontaneous curvature of MTs and shifting the distribution of protofilament number [190]. This phenomenon suggests that tau can also act as allosteric regulator, influencing the binding of additional MAPs on the outer surface of MTs [190].

On the contrary, MAP2 localizes to dendrites of mature neurons [191] where MT stabilization and growth are required. MAP2 is distributed at the crossbridge between dendritic MTs or between dendritic MTs and other cytoskeletal components [192,193]. Because of the association of MAP2 with MTs, they become stabilized and their stiffness is increased, thus giving rise to longer polymers [194,195,196,197]. MAP2 depletion inhibits neurite formation and outgrowth of cerebellar macroneurons and carcinoma cell lines [198] and disrupts MT spacing and dendritic arbor complexity in the mouse brain [199,200].

Both MAP2 and tau contain a great number of serines and threonines, and their biological activities are regulated by the degree of their phosphorylation at these sites. Low levels of phosphorylation do not interfere with their binding to MTs while high levels of phosphorylation decrease their association. As we will discuss in the next section, this phenomenon has important pathological implications in the case of tau: hyperphosphorylation of tau make this protein more susceptible to aggregating into insoluble inclusions that destabilize MTs and make neurons more vulnerable and prone to degenerating.

## 5. Microtubule Dysfunction and Neurodegeneration

The impairment of MT functions is considered the central physiopathological mechanism of several neurodegenerative diseases. In this section, we will briefly describe how abnormalities in MT regulative pathways impair MT properties and functions, leading to neurodegeneration.

### 5.1. Tubulin Dysfunction: Primary Tubulinopathies

As previously reported, mutations in tubulin genes are associated with a wide spectrum of neurological disorders collectively known as “tubulinopathies” and mostly characterized by cortical malformations. Despite tubulinopathies primarily causing neurodevelopmental diseases, in the last ten years, several studies have shown that specific tubulin isotypes or even mutations in specific residues of tubulin isotypes can also cause neurodegenerative profiles. How tubulin mutations impair tubulin functions and neuron activities, causing neurodegeneration or neurodevelopmental defects, is still not clear and certainly represents an intriguing area of investigation. To date, four tubulin isotypes, TUBB3, TUBB2A, TUBA4A, and TUBB4A, have been associated with neurodegenerative phenotypes [71,72,94,95,96,97,98,101,102,103,201,202] (Table 1).

TUBB3 was the first tubulin isoform to be associated with a neurodegenerative phenotype characterized by adult-onset axonal sensory motor polyneuropathy [71,72].

To date three neurodegeneration-causing mutations (D417N, D417H, and E410K) have been identified in TUBB3; all disrupt the MT interaction with kinesin motor proteins [71,203], inducing the accumulation of kinesins in axon tips and perturbing the axonal transport of synaptic vesicles and mitochondria in neurons [71,101]. Both D417 and E410 are highly conserved residues located in the H12 helix of β3-tubulin, and the substitutions affecting these amino acid positions are predicted to change the net negative charge of this domain, which is critical for the binding of kinesin motor proteins [71,101]. 

Interestingly, the D417N substitution has also recently been identified in TUBB2A in a patient affected by progressive spastic ataxia and sensory motor axonal neuropathy [94]. 

Consistent with such a critical role of D417 residue in kinesin binding and axonal transport, co-immunoprecipitation experiments have shown that the mutant TUBB2A was unable to bind KIF1A, a neuron-specific kinesin required for transport of synaptic vesicle precursors. Moreover, in vitro experiments have demonstrated that D417N substitution in TUBB2A causes cell cycle defects, impairing MT dynamics in the mitotic spindle [94]. Similar defects have previously been described in several neurodegenerative disorders [204,205,206,207,208] and were proposed to generate defective aneuploid neurons that are more prone to degeneration [209,210]. 

Moreover, in 2014, a large-scale analysis of exome-wide variant burdens involving 363 cases of familial amyotrophic lateral sclerosis (FALS) identified six pathogenic variants in TUBA4A (G43V, R215C, R320C, R320H, A383T, and W407X) [95]. All mutations disrupt MT dynamics and stability through a dominant-negative mechanism. Furthermore, the R320C/H, A383T, and W407 mutations in TUBA4A were also reported to impair α–β heterodimer formation [95]. Interestingly, the W407X mutation, that removes the last 41 amino acids of the C-terminal domain of TUBA4A, completely abolishes the incorporation of tubulin into MTs. Mutant TUBA4A harboring the W407X substitution shows aggregation propensities and, analogous to other ALS-associated mutant proteins, forms small cytoplasmatic inclusions. The biological relevance of these aggregates is not yet clear; it was proposed that these inclusions may act in trapping MAPs into aggregates or overloading the ubiquitin proteasome system [95].

Instead, depending on their position, mutations in *TUBB4A* may cause three neurodegenerative disorders: (i) hypomyelination with atrophy of the basal ganglia and cerebellum (H-ABC), a rare hypomyelinating disease of infancy and childhood [98,99,100,201,211,212,213,214,215,216,217,218] (ii) hereditary spastic paraplegia [96,97,98]; (iii) DYT4 with whispering dysphonia and normal MRI [96,97,98].

To date, 23 H-ABC-linked *TUBB4A* mutations have been described. In patients harboring these mutations, a lack of age-appropriate myelin formation is accompanied by progressive neuronal atrophy of specific brain regions, namely the basal ganglia and cerebellum. The most common H-ABC-related mutation is the D249N substitution [98,201,202]. D249 is located within the T7 loop and is predicted to form a salt bridge with the R2 located in the amino-terminal domain of TUBB4A. This interaction is critical for the positioning of the T7-loop and allows it to contact the GTP of α-tubulin in the α–β heterodimer [201]. 

According to the neuropathological phenotype characterized by the loss of putaminal neurons and oligodendrocytes [98,99,201,211,212], D249N mutation has been reported to affect both neuronal and oligodendroglial precursor cells in vitro and in a H-ABC mouse model [219,220].

Moreover, Vulinovic and colleagues have demonstrated that D249 substitution caused greater incorporation of the mutant TUBB4A into MTs and reduced the MT affinity for the kinesin KIF5 [221]. Accordingly, engineered D249N iPSCs present impaired mitochondrial axonal transport, characterized by reduced mitochondrial movement and increased mitochondrial speed, with the latter probably the consequence of a compensatory mechanism [221].

The H190Y and D353V mutations in TUBB4A have instead been described in patients affected by congenital-onset spastic paraplegia, which can occur concurrently with mild hypomyelination [101,102,103]. Functional data associated with these two mutations are still not reported.

Lastly, the heterozygous mutations p.R2G and p.A271T in TUBB4A have been associated with DYT4 [96,97].

p.R2G affects a highly conserved residue in the amino-terminal β-tubulin tetrapeptide Met–Arg–Glu–Ile (MREI), an autoregulatory domain that modulates the stability of polysome-bound tubulin messenger RNAs [222]. Yen and colleagues have been previously demonstrated that p.R2G substitution abrogates the MREI autoregulatory ability to destabilize β-tubulin mRNA, affecting the balance of tubulin subunits and interfering with MT proper assembly [222]. 

Differently from the previous study of Yen et al., Lohmann and colleagues detected a consistent decrease in mutant *TUBB4A* transcript levels of different cell types obtained from a patient harboring the p.R2G mutation [97]. Further studies will be necessary to explain this discrepancy and describe the mechanisms through which p.R2G mutation affects MT biochemical and functional properties. Recently, both p.R2G and p.A271T substitutions were reported to diminish TUBB4A ability to interact with α-tubulin, altering the MT network and inhibiting the neuronal growth process [223].

Overall, these findings highlight the specialized behavior of tubulin isotypes: depending on the affected isoforms and their location, tubulin mutations impair different MT functions, affecting specific cell types.

### 5.2. Tubulin PTM Imbalance

Several findings have shown that the disruption or dysregulation of PTMs are implicated in neurodegeneration processes [224,225,226,227,228,229,230,231,232].

Imbalance of α-tubulin acetylation and concomitant axonal transport defects have been found to be linked to different neurodegenerative diseases [224,225,226,227,228].

In the brains of patients affected by Alzheimer’s disease (AD), the levels of acetylated α-tubulin were found to be reduced in neurons containing neurofibrillary tangles [228]. Moreover, the levels of the deacetylating enzyme HDAC6 have been reported to be significantly increased in relevant degenerating brain regions of AD patients and AD animal models [229,230,231]. 

The loss of HADC6 in transgenic APP/PS1 mice, a model of AD, has been reported to restore the level of α-tubulin acetylation and ameliorate AD cognitive pathology [230]. 

Similarly, null mutants of HDAC6 rescue tau-mediated MT defects in both muscle and neurons of a drosophila model of tauopathies in which human tau is overexpressed in muscle cells [232].

Reduced levels of tubulin acetylation and defective axonal transports have also been described in motor neurons of patients affected by Charcot–Marie–Tooth disease (CMT) [227], a hereditary motor and sensory peripheral neuropathy. Accordingly, decreased levels of α-tubulin acetylation as well as axonal transport defects were also reported in peripheral neurons of mutant HSPB1-transgenic mice, a murine model of CMT2F [224]. 

Interestingly, in these cases, HDAC6 inhibition has also been reported to reverse mitochondrial axonal movement defects of CMT motor neurons [227] and to improve the phenotype of mutant SOD1 mice [225].

Reduced levels of acetylated tubulin have also been observed in the brains of patients affected by Huntington disease (HD), and the pharmacological treatment of mouse striatal cells derived from transgenic HD mice with compounds that increment tubulin acetylation stimulates MT-dependent transport of BDNF and prevents the alterations observed in HD mutant cells [131].

Imbalance of acetylated tubulin together with tyrosinated tubulin has also been observed in brain tissues of *Parkin* knockout mice which exhibit motor dysfunction and dopaminergic neuronal loss [233]. Dopaminergic neurons of young age brain tissues show an early accumulation of tyrosinated tubulin and decreased levels of acetylated tubulin, while at the later ages, the situation is overturned [234].

Tubulin glutamylation has been recently found to be involved in neurodegeneration as well.

The excessive polyglutamylation of tubulin has been linked to the rapid degeneration of Purkinje cells in *pcd* mice which present an inactivating mutation in the deglutamylating enzyme CCP1 [235] and in *Ccp1^flox/flox^* L7-*cre* mice in which the *CCP1* gene is specifically deleted in Purkinje cells [236].

Both mice displayed massive cerebellar atrophy, abnormally accumulate glutamylated tubulin in degenerating neurons, and display aberrant mitochondria axonal transport [237].

In *Ccp1^−/−^* neuron, hyperglutamylation of tubulin was reported to impair motility of several cargo items such as mitochondria, lysosomes, Lamp1-positive endosomes, and BDNF vesicles [238].

Importantly, Magiera and colleagues have demonstrated that hyperglutamylation of tubulin, rather than MT-severing enzymes that specifically target polyglutamylated MTs, is responsible for neurodegeneration since the concomitant deletion of the main brain glutamylase enzyme TTLL1, but not of the severing enzyme spastin, is able to prevent neuronal cell death [236].

In 2018, 6 loss-of-function mutations in human *CCP1* were described for 13 patients affected by infantile-onset progressive neurodegeneration mainly involving the cerebellum, spinal motor neurons, and peripheral nerves. The disease-associated mutation lead to the absence of functional CCP1, determining the accumulation of polyglutamylated tubulin and the loss of Δ2-tubulin [239].

Interestingly, CCP1-deficient mice recapitulate several key features of the human disease, including abnormal tubulin polyglutamylation, cerebellar atrophy, and the involvement of spinal motor neurons and peripheral nerves [239].

Recently Vu and colleagues have reported high levels of polyglutamylated and Δ2 tubulin in a kainate-induced epileptic seizure mouse model and the slow-developing AD brain of patients [240].

### 5.3. MAP Dysfunction 

Several findings have widely demonstrated that dysfunctions in MT-interacting proteins trigger neurodegeneration via defective regulation of MTs [241,242,243].

AD is probably the best example of neurodegenerative disease stemming from MAP dysfunction.

It is a member of a family of neurodegenerative disorders called tauopathies [244]; they are diseases of the nervous system in which tau becomes abnormally phosphorylated and accumulates in insoluble inclusions within brain neurons and often glia [245,246]. These tau accumulations are referred to as neurofibrillary tangles when found in the neuronal soma, and neuropil threads when found in dendritic processes [247,248]. Unlike classic tauopathies, AD is also characterized by the extracellular accumulation of β-amyloid (αβ) as senile plaques in specific brain regions [249]. Several lines of evidence indicate that αβ accumulation initiates tau phosphorylation [250,251,252,253,254]. Indeed, the exposure of primary neurons to oligomers of Aβ cause mislocalization of Tau in dendrites and the MT breakdown in dendrites invaded by Tau [254]. 

It has been demonstrated that Tau hyperphosphorylation promotes tau sequestration into neuronal inclusion and impairs MT stability [255,256].

Hyperphosphorylated tau isolated from the brain homogenate of AD patients is not able to promote MT assembly in vitro and its MT assembly activity is recovered only upon tau dephosphorylation with alkaline phosphatase treatment [257]. Moreover, hyperphosphorylated tau displays prion-like activity, sequestrating normal phosphorylated tau and other MAPs, such as MAP1 and MAP2, and destroying pre-assembled MTs in vitro [257,258]. 

To date, more than 40 phosphorylation sites have been identified in tau protein isolated from AD brain [259] and, depending on their position, have been reported to have a different impact on the pathogenic role of tau. In vitro kinetic studies have demonstrated that Ser199/Ser202/Thr205, Thr212, Thr231/Ser235, Ser262/Ser356, and Ser422 are the critical phosphorylation sites that convert tau to an inhibitory molecule that sequesters normal MAPs from MTs. Phosphorylation sites also seem to differently reduce tau affinity for binding MTs: phosphorylation of tau at Ser262, Thr231, and Ser235, for example, inhibits its MT affinity binding by ~35%, ~25%, and ~10%, respectively [259].

Transgenic mouse models of AD display fewer MTs [257,258,259,260,261], increased MT hyperdynamicity, axonal dystrophy, and reduced fast axonal transport [262,263]. The ectopic expression of human tau in *Drosophila melanogaster* results in decreased MT density, increased MT fragments, and increased amounts of satellite boutons at neuromuscular junctions [222]. Similarly, there is also evidence of MT deficits in the AD brain [228,256,264]. Ultra-morphometric analysis conducted on brain biopsies from AD patients documented a reduced MT density in pyramidal neurons [264,265]. Moreover, Zhang and colleagues [265] documented a decrease in the total level of α-tubulin and of α-tubulin-positive axonal processes in the neurons of AD patients.

In addition to AD, MT dysfunction has been considered an important contributor also to the pathogenesis of Parkinson’s disease (PD), a neurodegenerative condition characterized by the loss of dopaminergic neurons from substantia nigra and the presence in these cells of intracellular inclusions of α-synuclein (α-syn) [266].

Mutations in genes encoding the MT-interacting proteins Parkin, leucine-rich repeat kinase 2 (LRRK2), and α-syn indeed cause autosomal forms of parkinsonism [267].

By directly or indirectly binding tubulin, these proteins have been reported to modulate MT stability and function [268,269,270,271,272].

Mutations in *PRKN* gene, encoding the Parkin protein, are the most common cause of autosomal recessive juvenile parkinsonism and a major contributor to familial and sporadic early-onset PD [273,274]. Parkin is an E3 ubiquitin ligase that catalyzes the covalent attachment of ubiquitin to specific substrates, targeting them for proteasomal degradation [275]. This protein co-purifies with tubulin and was found in highly purified tubulin preparations [270]. Ren and colleagues have been reported that Parkin bound to α–β tubulin heterodimers with high affinity and that this interaction enhanced the ubiquitination and degradation of tubulins. In the same study, the authors demonstrated that Parkin colocalized with MTs in HEK293T cells and that the point mutations K161N, T240R, and C431F, associated with PD, abolished tubulin ubiquitination [268]. In 2005, Yang and colleagues reported that Parkin stabilized MT by binding three independent MT-binding domains (linker, RING1, and RING2) and that the overexpression of these domain as well as wild-type Parkin significantly attenuated colchicine-induced MT depolymerization in COS-7 cells [270]. Moreover, the authors demonstrated that the MT-binding activity of Parkin and its E3 ligase activity are independent since PD-linked mutations K161N, T240R, and C431F were able to impair its E3 ligase activity but not MT binding and stabilization [270]. Cartelli and colleagues have demonstrated that primary fibroblasts obtained from PD-affected patients exhibited reduced MT mass and higher MT destabilization and that overexpression of wild type Parkin restored the control phenotype [276]. Accordingly, experiments performed in both murine and human midbrain dopaminergic neurons showed that mutations or exon deletion in PRKN caused MT destabilization, abolishing the stabilizing effect of Parkin against MT-destabilizing toxins [277,278]. 

LRRK2 is another example of a microtubule-interacting protein associated with familial PD [279].

LRKK2 is a large multidomain protein and has been found associated with MTs [269].

LRRK2 was reported to co-immunoprecipitate with β-tubulin from both wild-type mouse brain and LRRK2-overexpressing HEK293T cells [280]. Moreover GFP-tagged LRRK2 was found to co-localize with β-tubulin in HEK-293 cells and the endogenous LRKK2 was found to colocalize with tubulin in primary hippocampal neurons [226,281].

Law and colleagues demonstrated that LRRK2 directly binds the carboxy-terminal domain of TUBB, TUBB4, and TUBB6 and that the binding specificity is determined by lysine 362 and alanine 364 of these β-tubulin isotypes. Interestingly, this interaction takes place at the luminal MT surface close to the K40 acetylation site of α-tubulin, a residue exposed in dynamic MT populations which present a more open and flexible conformation [269]. Consistent with this finding, endogenous LRRK2 displayed preferential localization to dynamic MTs within the growth cone [269]. Accordingly, mouse embryonic fibroblasts derived from LRRK2 knockout mice displayed increased MT acetylation, indicating that LRRK2 may interfere with tubulin acetylation [269]. 

LRRK2 was further reported to phosphorylate β-tubulin purified from bovine brain [280]. In vitro co-incubation of bovine brain tubulins with LRRK2 increased MT formation and stability in the presence of MAPs. Consistent with these data, Gillardon and colleagues detected higher levels of soluble tubulin in brain lysates from *LRRK2* knockout mice and shorter neurites in cell cultures of *LRRK2*-deficient neurons [280]. However, MacLeod and colleagues demonstrated that LRRK2 depletion in cortical neurons increased neurite length after 2 weeks [282]. Hence, further experiments will be necessary to explain the nature of LRRK2 and tubulin interaction and clarify this discrepancy.

LRRK2 was recently proposed also to control tau release from MT by mediating its phosphorylation [283,284]. Indeed, in a PD mouse model carrying the P301L mutation in LRRK2, the expression of mutant LRRK2 significantly increased hyperphosphorylated tau deposition [284].

By contrast, missense mutation and duplication and triplication of the SNCA gene encoding α-Syn underlies autosomal forms of parkinsonism [285].

α-Syn is a small, soluble unfolded protein that localizes predominantly to presynaptic terminals of neurons [286]. Although little is known about the mechanisms through which α-Syn acts in PD, different studies have recently proposed that this protein may act as a functional MAP by directly binding MTs [272,287] and regulating their stability. However, the effect of this interaction has been quite controversial, since different studies have reported that α-Syn, promotes MT stabilization, while others suggested the opposite effect [287,288].

Recently, Cartelli and colleagues have demonstrated that α-Syn interacts with pre-formed MTs in co-sedimentation experiments, co-purifies with brain tubulin and forms a specific complex with the tubulin α2β2 tetramer [271]. In the same study, the authors demonstrated that upon interaction with the tubulin α2β2 tetramer, α-Syn acquires a helical structure and becomes able to govern multiple steps of MT assembly and dynamics, such as nucleation, growth rate, and catastrophe frequencies, in a purified system as well as in a neuronal cell model [271], and that the PD-linked point mutations p.A30P, A53T, and E46K corrupt these functions and lead to tubulin aggregation [271].

Moreover, very recently, Cartelli and Cappeletti demonstrated that α-Syn is also able to regulate the partitioning between tubulin dimers and MTs in differentiated PC12 cells [289].

## 6. Microtubule-Targeting Agents

The awareness that MT dysfunctions are associated with several neurodegenerative disorders has promoted, in the last 20 years, numerous efforts to search for therapeutic compounds to correct MT defects related to neurodegeneration. 

Currently, two main lines of intervention exist: the first is centered on drugs that promote MT stability and assembly by directly binding tubulin, while the second relies on drugs that target tubulin enzymes which impact on MT functional properties by modifying tubulin PTMs. In this section, we will briefly discuss the most relevant therapeutic compounds developed for each of the two lines of intervention (Table 2).

### 6.1. Microtubule Stabilizing Compounds

The concept of utilizing MT-stabilizing drugs for the treatment of neurodegenerative diseases was tested for the first time in a tau transgenic mouse model of tauopathies in which the mouse prion protein (PrP) drives the overexpression of the shortest human brain tau isoform, T44, in the neurons of the central nervous system (PrP T44 mouse). At 3 to 12 months old, Prp T44 mice exhibit tau pathology, including accumulation of tau inclusions and insoluble hyperphosphorylated tau, reduced MT numbers, and impaired fast axonal transport in the spinal motor neurons that project outside the blood–brain barrier [290].

Zhang and colleagues [290] addressed the therapeutic potential of the MT-stabilizer paclitaxel.

Paclitaxel is a member of the taxane family, and initially emerged as an anticancer drug [305]. 

It promotes MT assembly in vitro [306] by binding within the lumen of the MT at a site in the β-tubulin subunit which is commonly referred to as the taxane-binding site [307,308]. Paclitaxel binding prevents compaction at the tubulin dimer interface and results in more stable lateral interactions, reducing the stochastic switching of MTs between growth and shrinkage [309].

Intravenous administration of paclitaxel in PrP T44 mice restored axonal transport in spinal axons and reduced the motor phenotype [290]. Moreover, beneficial effects of paclitaxel have been mechanistically linked to the stabilization of MTs because its administration increased the levels of stable detyrosinated tubulin as well as the numbers of MTs in the ventral root axons of transgenic mice [290].

Despite these beneficial effects, paclitaxel turns out to be an unsuitable therapeutic candidate for diseases affecting the brain because of its limited brain bioavailability. Paclitaxel does not cross the blood–brain barrier and its effectiveness in improving the phenotype of PrP T44 mouse depends on its uptake at neuromuscular junctions and subsequent retrograde transport to the spinal cord [290].

More recent studies have focused on the therapeutic effect of the MT-stabilizer epithilone D (Epo D). Epo D is a taxol-derived small molecule which prevents MT disassembly by interacting with β-tubulin at the taxane-binding site. This compound is probably the most promising MT stabilizer due to its excellent blood–brain barrier penetration and prolonged brain retention [310]. Indeed, it is selectively accumulated in the central nervous system, reducing its unwanted effects in the peripheral nervous system or elsewhere in the body [310].

The treatment of different lines of tau mouse models with Epo D has improved a number of central nervous system outcomes, increasing MT density, axonal integrity, and neuronal survival [263,291,310]. Moreover, in both young and old PS19 tauopathy models in which tau pathology is developing or well established, Epo D reversed behavioral and cognitive deficits, cleared tau pathology, and curbed neuron loss [291]. Epo D has also recently undergone evaluation in the 1-methyl-4-phenyl-1,2,3,6-tetrahydropyridine (MPTP)-induced mouse model of PD [292]. MPTP-treated mice showed an impairment of axonal transport in dopaminergic axons and changes in tubulin PTMs that were normalized following the treatment with Epo D. Moreover, repeated daily administrations of Epo D attenuated the loss of nigral dopaminergic neurons induced by MPTP [292]. Epo D has also been reported to restore the levels of acetylated α-tubulin and rescue the MT-based intracellular transport of peroxisomes in stem cells derived from patients affected by hereditary spastic paraplegia [293]. Fan and colleagues have demonstrated that very low doses of Epo D restored peroxisome speeds by increasing the number of stable MTs, and ameliorated patient cell sensitivity to hydrogen peroxide [293].

In 2012, Epo D has progressed to phase 1b clinical testing, in short 2–3 months studies in AD and/or tauopathy patients (NCT 01492374). Unfortunately, no results have been published, and drug development efforts for AD have halted. However, a new trial was since started, and the results are expected shortly (NCT 01966666).

Additional brain-penetrant MT-stabilizing products have been tested in recent years. 

Triazolopyrimidines (TPDs) are members of the vinca alkaloids which interact with MTs at a site that is distinct from the taxane-binding site, and generally targeted by agents such as vinblastine. The TPD binding of MTs stabilizes the longitudinal contacts between tubulin subunits and prevents MT disassembly [311]. In 2018, Zhang and colleagues tested CNDR-51657, a prototype of TPD, on a PS19 transgenic mouse model of tauopathies which expresses the P301S mutant form of human tau and shows the progressive accumulation of neurofibrillary tangles with age [294]. This study has demonstrated that low doses of CNDR-51657 significantly improved MT density in hippocampal neurons and reduced axonal dystrophy with a resulting reduction in tau pathology. Importantly, no adverse effects were observed in compound-treated mice, including no change in white blood cell counts [294].

Recently, Fanara and colleagues [293] have reported that the MT-modulating agent noscapine attenuates the deficits in axonal transport and improves MT stability in a mutant SOD1 transgenic mouse model of ALS which manifests MT hyperdynamicity in axons from the spinal cord, sciatic nerve, and cortex [295]. The noscapine mechanism of action is still not completely clear; it has been reported to specifically bind to tubulin and modulate MT dynamics by reducing the growing and shortening rates and increasing the percentage of time that MTs spend in the attenuated state [295].

However, among non-taxane MT-stabilizing agents, davunetide (also known as NAP or CP201) has reached the most advanced clinical development status. It is an 8 aa peptide neuropeptide (NAPVSIPQ) derived from the activity-dependent neuroprotective protein [312]. Although still elusive, the mechanism of action of NAP is believed to involve MTs. Affinity chromatography coupled with mass spectrometry identified tubulin as the major NAP-binding proteins in neurons and glial cells. Moreover, NAP decorates MTs in cultured cells and has been reported to enhance in vitro MT assembly in the presence of nocodazole [313]. However, Yenjerla and colleagues demonstrated that increased concentrations of NAP failed to induce tubulin polymerization in a purified system [314]. NAP was first tested on ApoE knockout mice, a mouse model for AD, and was reported to improve its short-term memory [296]. Recent studies have demonstrated that this compound enhances axonal transport and improves cognitive performance in both mouse models of AD and ALS disease [297,315]. Interestingly, NAP is brain penetrant when administered either by intranasal delivery (AL-108) [316,317], intravenously (AL-208) [318], or intraperitoneally [319]. 

NAP represents a potential therapeutic target for tauopathies; Magen and colleagues have reported that NAP blocks phosphorylation of tau on Ser262, thus increasing MT affinity for tau [317]. It is therefore likely that reduction in hyperphosphorylated tau by NAP is central for its mechanism [320,321]. NAP is in a phase II trial (NCT 01056965) to assess its effects on mild cognitive impairment in AD. Earlier analysis showed that NAP was well tolerated by AD patients with no obvious side effects, and there was some improvement in individual memory tasks but not for composite memory score [298,299].

### 6.2. Tubulin PTM-Targeting Compounds

The second line of intervention points to restore specific tubulin PTMs by modulating the activity of enzymes that directly regulate their level. At present, the major approach points to increase the levels of tubulin acetylation, which have been found to be reduced in several neurodegenerative diseases, through the inhibition of HDAC6 and Sirt2, the enzymes responsible for the deacetylation of α-tubulin.

The use of compounds that inhibit the enzyme HDAC6 represent, at the moment, a particularly promising therapeutic strategy. A few HDAC6 inhibitors are available. These compounds have been tested in several cellular and animal models of neurodegenerative diseases exerting neuroprotection. However, it is important to specify that almost all these classes of compounds, in addition to inhibiting HDAC6, also exert their actions on other HDACs. Moreover, the same HDAC6, besides the action to deacetylate α-tubulin, participates in other cellular processes, such as the transport and autophagic clearance of misfolded proteins [322]. Thus, the effect of these inhibitors is not always specific for tubulin acetylation and may involve additional processes.

In this section, we will focus our attention only on the effects that these compounds exert on tubulin acetylation.

The first HDAC6 inhibitor to be investigated was the natural compound trichostatin (TSA) [323,324]. It is a non selective HDAC inhibitor that acts by binding to the zinc ion at the HDAC active site of most zinc-dependent HDACs, including HDAC6 [325]. Its in vitro supplementation has been reported to lead to the hyperacetylation of tubulin in mammalian cells [324]. The pharmacological effects of TSA have been well established in several neurodegenerative diseases of the central nervous system. TSA was tested in an LRRK2 Drosophila model of PD. Systematic administration of TSA fully restored fly locomotor behavior and increased tubulin acetylation, improving axonal transport both in vitro and in vivo [223]. The same study showed that in vitro TSA treatments of rat cortical neurons expressing mutant LRKK2 increase tubulin acetylation and restore axonal transport [226].

Moreover, Hanson and colleagues have recently demonstrated that TSA prevents the decrease in tubulin acetylation precluding axon fragmentation and axonal loss in mouse cortical neurons exposed to kainic acid, an excitotoxic agent commonly used to induce epilepsy in rodents [300].

Together with tubastatin A (TubA), a selective inhibitor of HDAC6, TSA was reported to correct mitochondria axonal transport defects caused by HSPB1 mutations and rescue the CMT phenotype of symptomatic mutant HSPB1 mice [218].

TubA is a synthetic compound, first tested in primary cortical neuron cultures where it was found to induce elevated levels of acetylated α-tubulin, but not histone, consistent with its HDAC6 selectivity [326]. In a transgenic mouse model of tau deposition, TubA administration was reported to increase α-tubulin acetylation in the brain and revert behavioral defects such as memory impairment and hyperactivity [301]. Further, TSA was reported to increase vesicular transport of brain-derived neurotrophic factor by increasing the acetylation of α-tubulin in striatal precursor cells of a HD model [131].

AGK2, by contrast, is a selective inhibitor of the deacetylase Sirt2. In vitro experiments have shown that AGK2 increases the acetylation levels of tubulin heterodimers purified from bovine brain. Moreover, AGK2 treatment results in a dose-dependent increase in acetylation of soluble tubulin monomers and polymerized microtubules of HeLa cells without affecting their viability [302]. Moreover, in this study, the authors demonstrated that AGK2 prevents dopaminergic cell death both in vitro and in a Drosophila model of PD [302].

AK7, an additional inhibitor of SIRT2, has also been reported to increase acetylation of α-tubulin [303]. AK7 treatment exerts neuroprotective effect in models of PD and prevents in vivo dopamine depletion and dopaminergic neuron loss in MPTP-treated mice [304]. Moreover, its administration improved motor function, extended survival, and reduced brain atrophy in two genetic mouse models of HD [304].

## 7. Conclusions

Within the cell, MTs are among the most complex structures both in terms of chemical and functional properties. This complexity is generated through the combinatorial action of regulative mechanisms which determinate the temporal and tissue expression of different tubulin isotypes, their chemical modification as well as their interaction with proteins and cellular effectors.

Alterations of these regulative mechanisms impair MT functions. It has been widely demonstrated that MT dysfunction can contribute to, or be the cause of neurodegenerative processes.

This observation has received great attention in the design of novel therapeutic approaches aimed to counteract neurodegeneration by rescuing MT alterations. Compounds that modulate MT stability or restore the levels of tubulin acetylation have provided several benefits in cellular and animal models of different neurodegenerative diseases and some of them have been translated into clinical trials. These results have aroused great enthusiasm, encouraging research in this field. However, a better understanding of MT dysfunctions is necessary, such as through the analysis of the dynamic state of MTs in different neurodegenerative diseases. Indeed, although MT-stabilizing compounds can restore MT stability, excessively high doses of these drugs can disrupt MT integrity, promoting its over-stabilization. Instead, regarding the use of inhibitors for tubulin deacetylases, these drugs have multiple targets, and there is the need to develop compounds with greater selectivity.

Overall, despite these limits, MT-focused therapy actually represents an innovative and promising approach for the treatment of neurodegenerative diseases and may help to prevent or delay neurodegeneration in patients, improving their quality of life.

## Figures and Tables

**Table 1:** List of tubulin mutations causing neurodegenerative tubulinopathies.

Gene	Nucleotide Change	Amino Acid Change	Phenotype	References
***TUBB3***	c.1249G > A	D417N	Polyneuropathy without CFEOM3	[71,72]
***TUBB3***	c.1249G > C	D417H	CFEOM3	[71]
***TUBB3***	c.1228G > A	E410K	CFEOM3	[71]
***TUBB2A***	c.1249G > A	D417N	Progressive spastic paraplegia, polyneuropathy and ataxia	[94]
***TUBA4A***	c.128G > T	G43V	FALS	[95]
***TUBA4A***	c.958C > T	R320C	FALS	[95]
***TUBA4A***	c.643C > T	R215C	FALS	[95]
***TUBA4A***	c.959G > A	R320H	FALS	[95]
***TUBA4A***	c.1147G > A	A383T	FALS	[95]
***TUBA4A***	c.1221G > A	W407X	FALS	[95]
***TUBB4A***	c.4C > T	R2W	H-ABC	[98]
***TUBB4A***	c.5G > A	R2G	H-ABC	[98,99,100,101,102,103,104,105,106,107,108,109,110,111,112,113,114,115,116,117,118,119,120,121,122,123,124,125,126,127,128,129,130,131,132,133,134,135,136,137,138,139,140,141,142,143,144,145,146,147,148,149,150,151,152,153,154,155,156,157,158,159,160,161,162,163,164,165,166,167,168,169,170,171,172,173,174,175,176,177,178,179,180,181,182,183,184,185,186,187,188,189,190,191,192,193,194,195,196,197,198,199,200,201,202,203,204,205,206,207,208,209,210,211,212,213]
***TUBB4A***	c.467G > T	R156L	H-ABC	[214]
***TUBB4A***	c.533C > G	T178R	H-ABC	[213]
***TUBB4A***	c.533C > T	T178M	H-ABC	[202]
***TUBB4A***	c.533C > G	T178R	Severe H-ABC	[215]
***TUBB4A***	c.538G > A	V180M	H-ABC	[216]
***TUBB4A***	c.730G > A	G244S	H-ABC	[98,217]
***TUBB4A***	c.731G > T	G244V	H-ABC	[98,202]
***TUBB4A***	c.731G > A	G244D	H-ABC	[202]
***TUBB4A***	c.745G > A	D249N	H-ABC	[98,201,202,213,218]
***TUBB4A***	c.785G > A	R262H	H-ABC	[213,216,218]
***TUBB4A***	c.968T > G	M323R	H-ABC	[98]
***TUBB4A***	c.974G > T	W325L	H-ABC	[216]
***TUBB4A***	c.1054G > A	A352T	H-ABC	[98]
***TUBB4A***	c.1061G > A	C354Y	H-ABC	[98]
***TUBB4A***	c.1099T > A/C	F367I	H-ABC	[98]
***TUBB4A***	c.1162A > G	M388V	H-ABC	[98,213]
***TUBB4A***	c.1163T > C	M388T	H-ABC	[98,202]
***TUBB4A***	c.1164G > A	M388I	H-ABC	[98,217]
***TUBB4A***	c.1181T > G	F394C	H-ABC	[217]
***TUBB4A***	c.1228G > A	E410K	H-ABC	[211,213]
***TUBB4A***	c.544C > A	P182T	H-ABC	[202]
***TUBB4A***	c.568C > T	H190Y	Congenital-onset spastic paraplegia and mild hypomyelination	[101,102]
***TUBB4A***	c.1064A > T	D353V	Early-onset progressive spastic paraplegia	[103]
***TUBB4A***	c.4C > G	R2G	DYT4	[96,97]
***TUBB4A***	c.811G > A	A271T	DYT4	[97]

**Table 2:** List of most relevant MT-targeting compounds.

Drug	Target	Function	Effects	Model	References
**MT-Stabilizing Compounds**					
**Paclitaxel**	MTs	Stabilizes MTs	Restores axonal transport in spinal axons and reduces the motor phenotype, increases the levels of stable detyrosinated tubulin as well as the numbers of MTs in the ventral root axons	PrP T44 mouse	[290]
**Epo D**	MTs	Stabilizes MTs	Reverses behavioral and cognitive deficits, clears tau pathology and curbs neuron loss	Young and old PS19 mouse	[291]
**Epo D**	MTs	Stabilizes MTs	Attenuates nigrostriatal degeneration	MPTP-treated mice	[292]
**Epo D**	MTs	Stabilizes MTs	Restores the levels of acetylated α-tubulin, restores peroxisome speeds, and ameliorates patient cell sensitivity to hydrogen peroxide	iPSCs derived from patients affected by hereditary spastic paraplegia	[293]
**Epo D**	MTs	Stabilizes MTs	Not reported	Clinical trial	NCT 01492374 NCT 01966666
**CNDR-51657**	MTs	Stabilizes MTs	Improves MT density in hippocampal neurons and reduces axonal dystrophy with resulting reduction in tau pathology	PS19 mice	[294]
**Noscapine**	MTs	Stabilizes MTs	Attenuates the deficit of axonal transport and improves MT stability	Mutant SOD1 mice	[295]
**NAP**	May involve MTs	May prevent MTs disruption	Improves short-term memory	ApoE knockout mice	[296]
**NAP**	May involve MTs	May prevent MTs disruption	Increases soluble tau and reduces tau hyperphosphorylation at Ser202	DM-tau mice	[297]
**NAP**	May involve MTs	May prevent MTs disruption	Improves the individual memory tasks	AD patients	NCT 01056965 [298,299]
**Tubulin PTMs targeting compounds**					
**TSA**	HDAC6 (not selective)	Increases tubulin acetylation	Restores fly locomotor behavior and increases tubulin acetylation improving axonal transport	LRRK2 drosophila model of PD	[226]
**TSA**	HDAC6 (not selective)	Increases tubulin acetylation	Preventing axon fragmentation and axonal loss	Mouse cortical neurons expose to kainic acid	[300]
**TSA**	HDAC6 (not selective)	Increases tubulin acetylation	Increases vesicular transport of brain-derived neurotrophic factor by increasing acetylation of α-tubulin	Striatal precursor cells of HD model	[131]
**TSA** **TubA**	HDAC6	Increases tubulin acetylation	Corrects mitochondria axonal transport defects	HSPB1 mice	[216]
**TubA**	HDAC6	Increases tubulin acetylation	Reverts the behavioral defects such as memory impairment and hyperactivity	rTg4510 mice	[301]
**AGK2**	Sirt2	Increases tubulin acetylation	Prevents dopamine depletion in disease models of PD	Drosophila model of PD	[302]
**AK7**	Sirt2	Increases tubulin acetylation	Prevents dopaminergic neuron loss in vivo in MPTP-treated mice, improves motor function, extends survival, and reduces brain atrophy in HD mice	MPTP mice, R6/2 mice, and HD knock-in mice	[303,304]

## Abbreviations

MT	microtubule
MAPs	MT-associated proteins
PTMs	post-translational modifications
MTOC	MT-organizing center
CFMO3	congenital fibrosis of the extraocular muscles type 3
DYT4	torsion dystonia type4
ALS	amyotrophic lateral sclerosis
K40	lysine 40
HDAC6	histone deacetylase 6
TTL	tubulin tyrosine ligase
CCP	cytoplasmic carboxypeptidases
TIP	end-tracking protein
FALS	familial amyotrophic lateral sclerosis
H-ABC	atrophy of the basal ganglia and cerebellum
MREI	tetrapeptide Met–Arg–Glu–Ile
AD	Alzheimer’s disease
CMT PD	Charcot–Marie–Tooth disease Parkinson’s disease
αβ α-syn LRRK2 PrP	β-amyloid α-synuclein leucine-rich repeat kinase 2 mouse prion protein
Epo D	epithilone D
MPTP	1-methyl-4-phenyl-1,2,3,6-tetrahydropyridine
TPD	triazolopyrimidines
NAP	davunetide
TSA	trichostatin
TubA	tubastatin A
